# Comparison of Laparotomy and Percutaneous Intraperitoneal Lavage on Survival in a Cecal Slurry Peritonitis Model in Preterm Mice

**DOI:** 10.24546/0100504291

**Published:** 2026-05-20

**Authors:** YOSHITOMO SAMEJIMA, SHOHEI YOSHIMURA, HARUNORI MIYAUCHI, YUICHI OKATA, YUKO BITOH

**Affiliations:** 1 Division of Pediatric Surgery, Department of Surgery, Kobe University Graduate School of Medicine, Kobe, Japan

**Keywords:** Bacterial peritonitis, Cecal slurry, Laparotomy, Preterm infants, Percutaneous peritoneal drainage

## Abstract

We aimed to compare survival rates in a 4-day-old mouse model of cecal slurry (CS)-induced bacterial peritonitis managed by laparotomy (LAP) or percutaneous peritoneal lavage (PPL). Four-day-old pups received intraperitoneal injections of CS to induce severe (3.0 g/gBW) or mild (1.0 g/gBW) peritonitis. Two hours later, pups underwent either LAP or PPL. Seven-day survival rates, perioperative body temperature (BT) changes, and postoperative ascites and plasma cytokine levels were assessed. In the severe peritonitis group, the 7-day survival rate tended to be higher in pups who underwent PPL than in those who underwent LAP (36.4% vs. 9.1%, *p* = 0.069). In the mild peritonitis group, survival rates were similar between treatments (83.3% vs. 72.7%, *p* = 0.53). The median perioperative BT change was significantly smaller in pups who underwent PPL for both the severe (−1.2°C vs. vs. −2.6°C, *p* < 0.01) and mild (−0.2°C vs. −2.9°C, *p* < 0.01) peritonitis groups. Postoperative tumor necrosis factor levels were significantly lower in the PPL group compared with the LAP group under mild peritonitis (202 pg/ml vs. 443 pg/ml, *p* = 0.034). We established a novel 4-day-old PPL mouse model and identified PPL as a potential minimally invasive alternative that might help prevent perioperative hypothermia in the fatal peritonitis.

## INTRODUCTION

Intestinal perforation results in peritonitis in preterm infants. This perforation comprises surgical necrotizing enterocolitis (NEC) and spontaneous intestinal perforation (SIP). Despite advancements in neonatal intensive care and surgical techniques, the mortality rate for preterm peritonitis remains at approximately 30% (1, 2). Initial treatment strategies for preterm peritonitis include laparotomy (LAP) or percutaneous peritoneal lavage (PPL); however, which approach could improve survival rates remains controversial (2–6). Since PPL is often chosen for managing infants with severe peritonitis, both peritonitis severity and infant’s overall condition are critical factors for determining the optimal intervention. Furthermore, in preterm and low-birth-weight infants with poor preoperative status, LAP may impose a higher surgical burden. There is still limited evidenced data on comparing the impact of different techniques, LAP, and PPL, in preterm infants with severe peritonitis. The cecal slurry (CS) mouse model, introduced by Wynn et al. in 2007, induces sepsis through intraperitoneal injection of adult mouse fecal suspension (7). Fujioka et al. showed that using 4-day-old mice in this model can approximate conditions in human preterm infants (8). Our group has previously reported on survival rates, cytokine levels, and temperature changes in a LAP-based CS model (9). In this study, we developed a novel mouse model employing PPL and aimed to compare survival outcomes between LAP and PPL in this mouse model with severe CS-induced bacterial peritonitis.

## MATERIALS AND METHODS

### Animals

Six 8-week-old FVB/NJc1 mice (adult mice) were purchased from CLEA Japan Inc. (Tokyo, Japan). The mice were maintained under controlled temperature (21–23°C), humidity (30–70%), and day/night lighting conditions (12-hour light/12-hour dark cycle). The mice had free access to standard chow for rodents and water. All pups remained in the same environment as their mothers throughout the experimental period. Pups were individually randomized to minimize bias within each litter. At least three litter groups were used in survival experiments.

### Preparation of CS solution

Using adult mouse cecum, the CS stock solution was prepared as previously described and stored in 1 mL aliquots at −80°C until use (7, 10, 11). For induction of bacterial peritonitis, aliquots were thawed at room temperature.

### Defining CS concentrations for severe and mild peritonitis

Previous studies indicate a dose-dependent reduction in survival with increasing CS concentration (8). Various CS volumes were administered intraperitoneally to 4-day-old mice to establish two groups: a severe peritonitis group (<50% 7-day-survival) and a mild peritonitis group (100% 7-day-survival); these groups were then used as controls.

### Establishing LAP and PPL models

Severe or mild peritonitis was induced in 4-day-old pups using previously determined CS doses. Two hours later, after checking for redness of the abdominal wall and peritonitis, either LAP or PPL was performed under anesthesia with isoflurane until reflexes were abolished; thereafter, the pups were placed in the supine position.

In the LAP group, a 5-mm transverse incision was made in the upper abdominal wall using scissors, and the abdomen was opened. The peritoneal cavity was irrigated with 500 μL of 25°C sterile normal saline at room temperature for 1 min, then drained using gauze. The abdominal wall was closed with two sutures of 6-0 PDS Plus (Ethicon Inc., Somerville, NJ, USA) (9).

**Figure 1:**
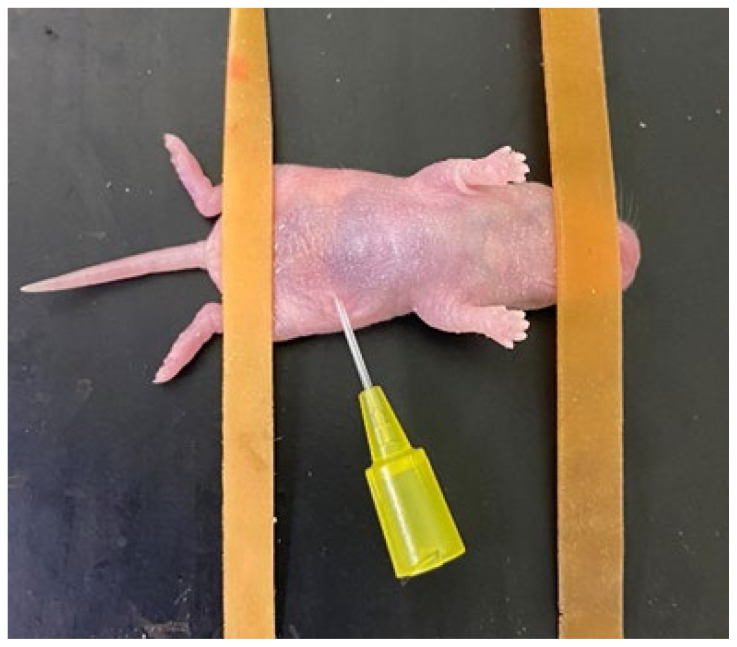
PPL model in a preterm peritonitis mouse. Under inhalation anesthesia, a 24-gauge intravenous catheter was inserted into the left upper quadrant of the abdomen. PPL, percutaneous peritoneal lavage.

In the PPL group, a 24-gauge Jelco® clear, non-radiopaque intravenous catheter (Smith Medical Japan, Tokyo, Japan) was inserted into the left upper quadrant, leaving the outer sheath in place (Figure 1). The peritoneal cavity was irrigated with 500 μL of 25°C sterile normal saline at room temperature; the fluid was removed using gauze before the catheter was withdrawn.

Immediately post-procedure, pups were returned to their mother’s cages and observed until spontaneous breathing and body movements fully recovered. Their postoperative course was then monitored. We defined the LAP and PPL groups in severe and mild peritonitis as 3-LAP, 3-PPL, 1-LAP, and 1-PPL groups, respectively. Furthermore, we randomized the four groups to be placed in their respective litters.

### Evaluation of postoperative survival and body weight (BW) changes

Postoperative survival was monitored for seven days in both LAP and PPL groups (severe and mild peritonitis). Preoperative and 24-hour postoperative BW were recorded, and the 24-hour BW change rate (%BW change) was calculated.

### Evaluation of perioperative body temperature (BT) changes

To assess the impact of different surgical techniques and peritonitis severity, BT was measured immediately before and after surgery; the perioperative BT change was calculated. BT was taken at the xiphoid process using a non-contact infrared thermometer (CUSTUM Co., Ltd., Tokyo, Japan) as previously described (9, 12).

### Measurement of postoperative ascites and plasma cytokines

Two hours after surgery, the pups were deeply anesthetized with isoflurane. Ascites and plasma were collected for each group. Sterile phosphate-buffered saline (BW × 40 μL) was injected intraperitoneally with a 28G microsyringe, and ascites was collected and stored at −80°C until analysis. Blood samples were taken via cardiac puncture in the superior thoracic aperture and mediastinum using a 28G needle syringe pre-treated with 1,000 U/mL heparin, kept on ice, and centrifuged at 4°C, 1,200 g for 15 min (Sorvall Legend Micro 21R, Thermo Fisher Scientific, Waltham, MA, USA). The plasma supernatant was stored at −80°C until analysis. These pups were euthanized immediately after sample collection.

Postoperative intra-abdominal and systemic inflammation were evaluated by measuring interleukin (IL)-12p70, tumor necrosis factor (TNF), interferon-γ (IFN-γ), monocyte chemotactic protein-1 (MCP-1), IL-10, and IL-6 in ascites and plasma using a BD Cytometric Bead Array Mouse Inflammation Kit (BD Biosciences, San Jose, CA, USA) and flow cytometry, according to the manufacturer’s recommended protocol. Analyses were performed on an LSRFortessa X-20 (BD Biosciences, San Jose, CA, USA) and data were processed with FCAP Array software (BD Biosciences, San Jose, CA, USA).

### Statistical analyses

Statistical analyses were performed using JMP® 15 (SAS Institute Inc., Cary, NC, USA) and GraphPad Prism version 10.2 (GraphPad Software, San Diego, CA, USA). Kaplan-Meier plots with log-rank tests were used for survival data. Perioperative BT changes, %BW changes, and cytokine concentrations were expressed as medians [minimum, maximum], and compared using the Mann-Whitney U test. Statistical significance was set at *p* < 0.05.

## RESULTS

### Determination of peritonitis severity

The severity of peritonitis was established based on 7-day survival rates following concentration-dependent CS injections, as previously described by our group (9). Consequently, the CS dose was set at 3.0 mg/gBW for severe peritonitis (3-sham group; 7-day survival rate: 42.9%) and at 1.0 mg/gBW for mild peritonitis (1-sham group; 7-day survival rate: 100%).

#### Postoperative 7-day-survival rates and 24-hour BW changes

**Figure 2:**
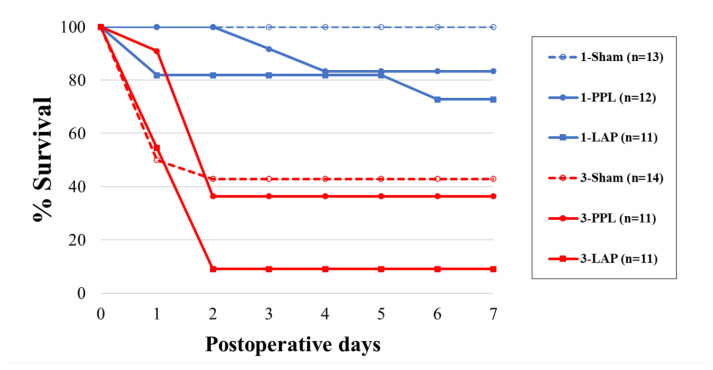
Seven-day survival rates for each group. The survival rates were 100% in 1-sham, 42.9% in 3-sham, 83.3% in 1-PPL, 72.7% in 1-LAP, 36.4% in 3-PPL, and 9.1% in 3-LAP. LAP, laparotomy; PPL, percutaneous peritoneal lavage.

In mice with severe peritonitis, the 7-day survival rate was 36.4% in 3-PPL and 9.1% in 3-LAP (*p* = 0.069). Under mild peritonitis, it was 83.3% in 1-PPL and 72.7% in 1-LAP (*p* = 0.53, Figure 2).

**Figure 3:**
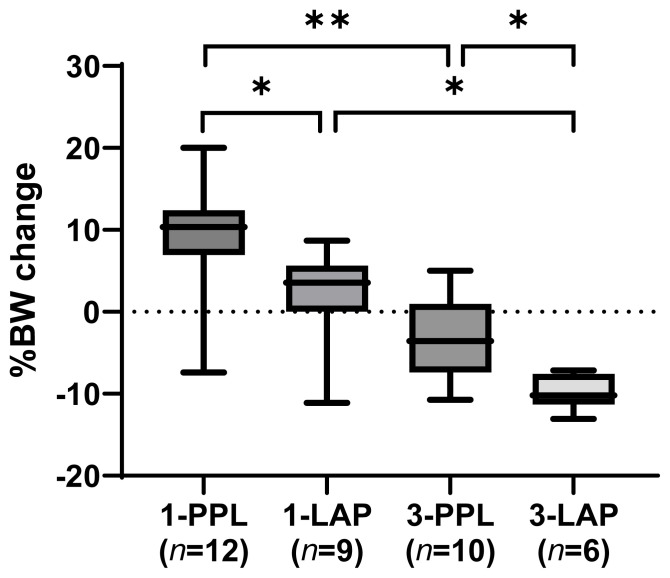
BW change rates in the first 24 hours postoperatively. The median [range] rates were 10.3% [−7.4, 20.0] in 1-PPL, 3.6% [−11.1, 8.7] in 1-LAP, −3.6% [−10.7, −5.0] in 3-PPL, and −10.2% [−13.0, −7.1] in 3-LAPP. *p < 0.05, **p < 0.01. BW, body weight; LAP, laparotomy; PPL, percutaneous peritoneal lavage.

All pups exhibited weight loss at 24 h postoperatively under severe peritonitis. The median BW change rates were −10.2% [−13.0, −7.1] in 3-LAP and −3.6% [−10.7, −5.0] in 3-PPL (*p* = 0.012, Figure 3). By contrast, under mild peritonitis, the median BW change rates were 10.3% [−7.4, 20.0] in 1-PPL and 3.6% [−11.1, 8.7] in 1-LAP (*p* = 0.013). Additionally, weight gain was significantly poorer in 3-LAP compared with 1-LAP (*p* = 0.011) and in 3-PPL compared with 1-PPL (*p* < 0.01).

### Perioperative BT changes

**Figure 4:**
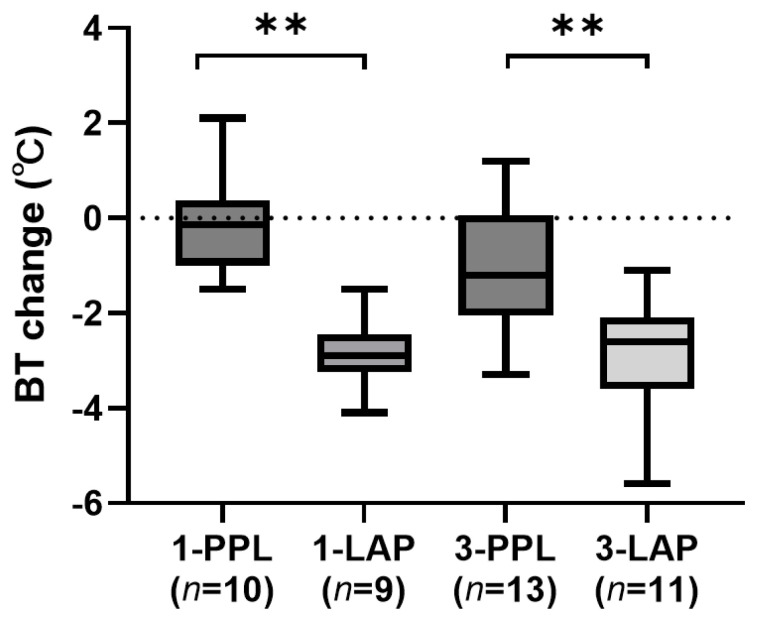
Perioperative BT changes. Median [range] changes were −0.2°C [−1.5, 2.1] in 1-PPL, −2.9°C [−4.1, −1.5] in 1-LAP, −1.2°C [−3.3, 1.2] in 3-PPL, and −2.6°C [−5.6, −1.1] in 3-LAP. **p < 0.01. BT, body temperature; LAP, laparotomy; PPL, percutaneous peritoneal lavage.

All pups undergoing LAP developed perioperative hypothermia regardless of peritonitis severity. Median perioperative BT changes were −2.9°C [−4.1, −1.5] in 1-LAP and −2.6°C [−5.6, −1.1] in 3-LAP (Figure 4). However, PPL significantly reduced hypothermia: median perioperative BT changes were −0.2°C [−1.5, 2.1] in 1-PPL (*p* < 0.01 vs. 1-LAP) and −1.2°C [−3.3, 1.2] in 3-PPL (*p* < 0.01 vs. 3-LAP).

### Cytokine concentrations in ascites and plasma 2 h after LAP and PPL in severe and mild peritonitis

#### Ascites (Figure 5)

Under severe peritonitis, the median ascites concentrations of IL-12p70, TNF, IFN-γ, MCP-1, IL-10, and IL-6 were not significantly different between the 3-PL and 3-LAP groups. Under mild peritonitis, the median ascites concentrations of these cytokines were not significantly different between the 1-PL and 1-LAP groups.

#### Plasma (Figure 6)

In mice with severe peritonitis, the median plasma concentrations of IL-12p70, TNF, IFN-γ, MCP-1, IL-10, and IL-6 were not significantly different between the 3-PPL and 3-LAP groups. Under mild peritonitis, the median plasma concentration of TNF was significantly different in 1-PPL vs. 1-LAP, 202 [162, 268] vs. 443 [390, 1258] pg/mL (*p* = 0.034). Other plasma cytokines were not significantly different between the 1-PPL and 1-LAP groups.

## DISCUSSION

In this study, we established a novel 4-day-old mouse model with PPL and compared its postoperative survival with that of a previously established LAP model under severe and mild peritonitis. The survival analysis indicated that LAP imposed greater surgical invasion in severe peritonitis, evidenced by a 7-day survival rate of 9.1% in the 3-LAP group versus 36.4% in the 3-PPL group. Conversely, under mild peritonitis, the 1-PPL and 1-LAP groups had similar 7-day survival rates of 72.7% and 83.3%, respectively, suggesting that PPL may serve as an effective alternative in life-threatening conditions. As noted above, given that PPL is more effective than LAP in the severe peritonitis model, it is reasonable to assume that the efficacy of PPL would be even more pronounced in a more severe peritonitis model. However, in a peritonitis model even more severe than the one used in this study, it is expected that all pups would die regardless of whether PPL or LAP is administered. From a clinical perspective, patients in such a condition are in a state where they lack the capacity to undergo surgery in the first place. In other words, the 1-Sham group corresponds to a systemic condition capable of tolerating both PPL and LAP; the 3-Sham group corresponds to a systemic condition capable of tolerating PPL but not LAP; and the even more severe peritonitis corresponds to a systemic condition incapable of tolerating either PPL or LAP.

Although LAP is the standard procedure for preterm infants with gastrointestinal perforation, it may exacerbate outcomes under severe peritonitis due to heightened surgical invasion, including insensible water loss, hypothermia from abdominal exposure, and amplified inflammatory responses (13). By contrast, PPL, a minimally invasive option, is sometimes chosen as a palliative option for critically ill infants with perforation. In our model, PPL prevented excess BW loss, diminished perioperative hypothermia, and helped avert the steep decrease in survival observed in the LAP group under severe peritonitis (mortality exceeding 50%).

Postoperative BW gain is a useful marker of hypovolemia in these surgical models. Normally, 4-day-old mice gain 20–30% of their BW within 24 hours. However, in mice with severe peritonitis, particularly in the LAP group, mice deviated significantly from this pattern. Moreover, BW change rate trends in each group reflected the survival outcomes, indicating that the combined metabolic and physiologic stress from severe infection and invasive surgery compromises recovery.

Significant perioperative hypothermia occurred in the LAP groups, regardless of peritonitis severity, supporting the conclusion that surgical invasiveness primarily drives intraoperative heat loss. This result aligns with previous reports describing radiation and evaporation during open abdominal procedures (9). In contrast, PPL markedly reduced hypothermia, suggesting that minimally invasive approaches better preserve thermoregulation. Hypothermia has wide-ranging adverse effects—such as platelet dysfunction, coagulopathy, and increased wound infection risk (14, 15)—and neonates, with their high surface-area-to-volume ratio, are particularly vulnerable (16). These results highlight how temperature management during less invasive procedures can critically influence outcomes in fragile preterm populations, and that combining minimally invasive surgery with adequate perioperative thermal support holds promise for improving survival and recovery.

We also measured postoperative pro- and anti-inflammatory cytokine concentrations to assess the local and systemic inflammatory burden associated with LAP versus PPL, but no significant differences were observed. A previous report from our group (9) similarly found no correlation between mortality and cytokine levels in ascites or plasma, highlighting the complex relationship between perioperative hypothermia, inflammation, and patient outcomes (17). Under severe peritonitis, the inflammatory load may mask subtle differences attributable to surgical techniques, and future work adjusting peritonitis severity might shed more light on how immune responses are affected by surgical invasion. Such insights could guide the design of improved surgical and perioperative management protocols for premature infants with gastrointestinal perforations. Further research using larger animal models and advanced monitoring technologies is warranted to investigate the thermoregulatory and inflammatory mechanisms more thoroughly.

Nonetheless, this study has several limitations. First, 4-day-old mice are extremely small, making establishment of venous line for drug administration difficult. Furthermore, intraperitoneal drug administration was considered likely to interfere with the experiment. For these reasons, drug administration was not feasible, which limited the implementation of standard perioperative care, such as ventilator management, precise circulatory management, and antibiotic therapy. Consequently, both the LAP and PPL groups were unable to surpass survival rates in untreated mice, and the direct therapeutic benefit of intraperitoneal lavage itself was not fully established. Second, although hypothermia was linked to mortality and delayed postoperative recovery, the exact mechanisms remain speculative. The sample size of mice for cytokine analysis is very small and might confound any conclusions that could be drawn from the data. Additional animal and clinical investigations are needed to clarify these processes.

In conclusion, a novel 4-day-old mouse model with PPL, which is a novel surgical approach for preterm infants with severe peritonitis, was successfully established. In our experiments, PPL served as a potential alternative by helping prevent excessive BW loss and perioperative hypothermia in severe, life-threatening peritonitis.

## Figures and Tables

**Figure 5:**
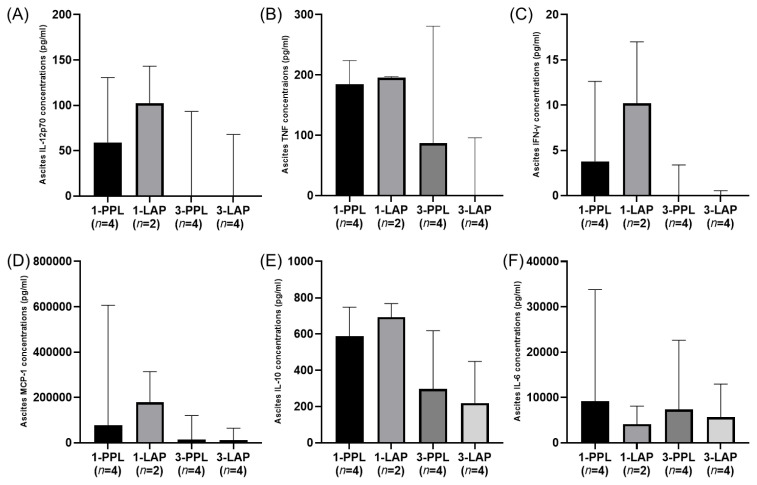
Ascites pro- and anti-inflammatory cytokine concentrations at 2 hours postoperatively: A, IL-12p70; B, TNF; C, IFN-γ; D, MCP-1; E, IL-10; and F, IL-6. IFN-γ, interferon-γ; IL-6, interleukin-6; IL-10, interleukin-10; IL-12p70, interleukin-12p70; LAP, laparotomy; MCP 1, monocyte chemotactic protein 1; PPL, percutaneous peritoneal lavage; TNF, tumor necrosis factor.

**Figure 6:**
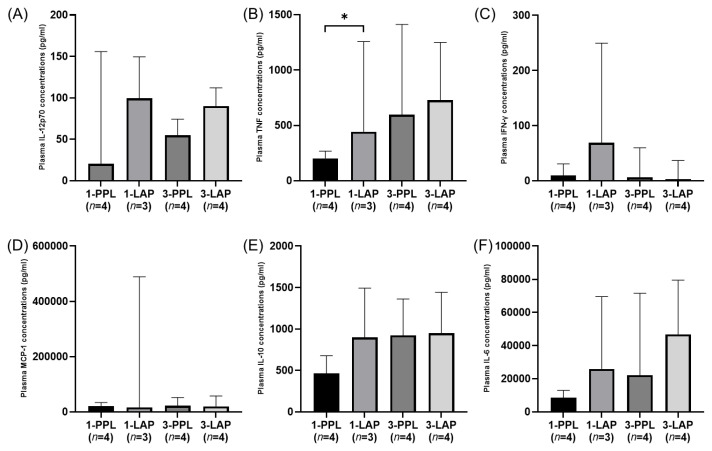
Plasma concentrations of pro- and anti-inflammatory cytokines at 2 hours postoperatively: A, IL-12p70, B, TNF; C, IFN-γ; D, MCP-1; E, IL-10; and F, IL-6. *p < 0.05. IFN-γ, interferon-γ; IL-6, interleukin-6; IL-10, interleukin-10; IL-12p70, interleukin-12p70; LAP, laparotomy; MCP-1, monocyte chemotactic protein 1; PPL, percutaneous peritoneal lavage; TNF, tumor necrosis factor.
